# Evolutionary immuno-genetics of endoplasmic reticulum aminopeptidase II (ERAP2)

**DOI:** 10.1038/s41435-023-00225-8

**Published:** 2023-11-04

**Authors:** Aroosha Raja, Jonas J. W. Kuiper

**Affiliations:** Department of Ophthalmology, Center for Translational Immunology, University Medical Center Utrecht, University of Utrecht, Utrecht, The Netherlands

**Keywords:** Antigen-presenting cells, Immunogenetics

## Abstract

Endoplasmic reticulum aminopeptidase 2 (ERAP2) is a proteolytic enzyme involved in adaptive immunity. The *ERAP2* gene is highly polymorphic and encodes haplotypes that confer resistance against lethal infectious diseases, but also increase the risk for autoimmune disorders. Identifying how ERAP2 influences susceptibility to these traits requires an understanding of the selective pressures that shaped and maintained allelic variation throughout human evolution. Our review discusses the genetic regulation of haplotypes and diversity in naturally occurring ERAP2 allotypes in the global population. We outline how these *ERAP2* haplotypes evolved during human history and highlight the presence of Neanderthal DNA sequences in *ERAP2* of modern humans. Recent evidence suggests that human adaptation during the last ~10,000 years and historic pandemics left a significant mark on the *ERAP2* gene that determines susceptibility to infectious and inflammatory diseases today.

## Introduction

*Endoplasmic reticulum aminopeptidase 2* (ERAP2) is a aminopeptidase located in the endoplasmic reticulum lumen that primarily functions in antigen processing for presentation by the class-I major histocompatibility complex (MHC-I) to initiate immune responses to infected cells [1]. Polymorphic variation in the form of single nucleotide variants (SNVs) in the *ERAP2* gene has been associated with increased susceptibility to chronic inflammatory disorders, such as *Crohn’s disease*, *birdshot chorioretinopathy*, and *ankylosing spondylitis*, as well as with protection against severe infections such as, pneumonia, as well as the bubonic plague (i.e., The *Black Death*) in history [2–5]. SNVs located in and downstream of the *ERAP2* gene form extended *ERAP2* haplotypes [6] that display dramatic differences in transcriptional expression, but there remains little understanding how these functionally distinct haplotypes arose and mechanistically modify disease susceptibility in the human population. While the *ERAP2* gene exhibits signatures of *long-standing balancing selection* [6], new studies indicate also recent genetic adaptation in Europeans. In this review, we summarise the evolutionary history of human *ERAP2* haplotypes and discuss how natural selection helped shape allelic diversity that influences susceptibility to chronic inflammatory disease and infection resistance.

## Biological functions of ERAP2

**Fig. 1:**
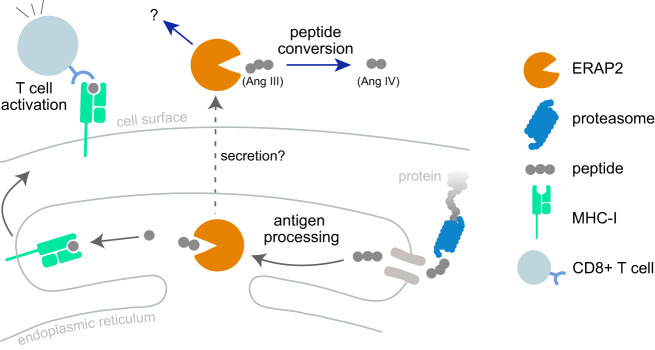
The biological functions of ERAP2. Protein fragments (peptides) generated by cellular proteasomes are transported into the endoplasmic reticulum. ERAP2 is an aminopeptidase that trims N-terminal amino acid residues from peptides in the endoplasmic reticulum to prevent or facilitate binding to the major histocompatibility complex class I (MHC-I). The MHC-I peptide complexes are then presented on the surface of the cell to CD8 + T cells, allowing the immune system to identify and respond to infected or abnormal cells. The ERAP2 enzyme has also been shown to remove amino acid residues from naturally occurring signalling peptides, including the conversion of angiotensin derivatives [Angiotensin II/III into IV], indicating a potential role for ERAP2 in renin-angiotensin signalling. Additional unidentified pathways may be regulated by extracellular or secreted ERAP2 proteins.

The biological functions of ERAP2 in health and disease have been reviewed elsewhere [7–9] and will be covered only briefly here. The *ERAP2* gene (43 kb in length) clusters together with the *ERAP1* (47 kb) and *LNPEP* (102 kb) on chromosome *5q15* which based on close sequence homology constitute a functional subfamily of mammalian zinc-containing aminopeptidases (or M1 metallopeptidases) that cleave N-terminal amino acids of bioactive and antigenic peptides [10, 11]. The evolutionary origins of these aminopeptidases remains uncertain, but others have speculated that *ERAP2* resulted from gene duplication from adjacent genes, such as *ERAP1* [6, 12]. As a M1 metallopeptidase, the 960 amino acid-long ERAP2 protein contains a GAMEN motif at amino acid position 334 (interacts with the N-terminus of peptide substrates) and a zinc-binding site encoded by amino acid motif HELAH (HEXXH-motif) at amino acid residues 370 (encoded by exon 6) [13, 14]. In vitro digestion analysis of purified ERAP2 has shown the capacity of the proteolytic enzyme to hydrolyze amino acid residues from the N-terminus of naturally occurring angiotensinergic and vascular inflammatory peptides, such as kallidin and angiotensin III (Fig. 1) [14]. Genome-wide association studies linking genetic variants in *ERAP2* to blood pressure [15] further support that ERAP2 may play a role in the renin-angiotensin system [16], however these functions remain largely speculative and require more investigation. Research on ERAP2 has been primarily focused on its role in antigen presentation [8, 9]. In the luminal site of the endoplasmic reticulum, ERAP2 is involved in the processing of N-terminal extended precursor peptides which determines their binding to MHC-I for presentation to CD8 + T cells at the cell surface (Fig. 1) [1, 14]. ERAP2 has been shown to generate the mature epitopes of immunogenic epitopes and directly enhance T cell recognition [17]. Mechanistically, ERAP2 shapes the immunopeptidome of the cell by the selective removal and indirect promotion of submotifs of peptides presented by MHC-I [18]. Due to its genetic association with several autoimmune diseases, ERAP2 may promote T-cell recognition of autoantigens, which contributes to destructive inflammation [7, 19, 20].

## Common genetic variation controls alternative splicing of *ERAP2*

**Fig. 2:**
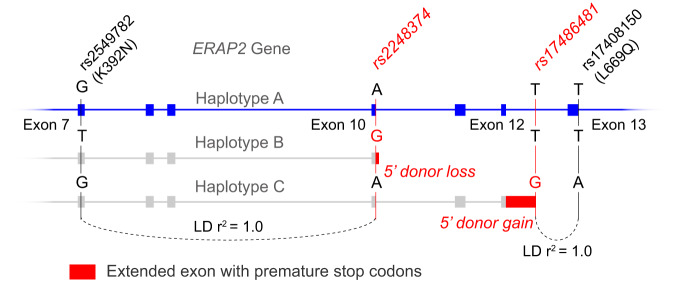
Two splice region variants mediate alternative splicing of the *ERAP2* gene. There are two common intronic single nucleotide variants (>1% in the global population) within *ERAP2* that mediate alternative splicing into three common haplotype groups (Haplotype A-C). With variant rs2248374 (Global allele frequency = 0.55) located downstream of exon 10, the G allele disrupts a donor splice site (5’ donor loss), resulting in an extended exon 10 which encodes multiple premature stop codons, resulting in mRNA decay and loss of full-length ERAP2 protein. The G allele of rs2248374 is in full linkage disequilibrium ([LD] from 1000 Genomes) with T allele of missense variant rs2549782 (392 N). A canonical splicing site motif (5’ donor gain) is introduced by the G allele of rs17486481 (global allele frequency = 0.02) located in the intronic region between Exon 12 and 13. Exon 12 will be extended and include premature stop codons that are prone to nonsense mediated decay. The G allele of rs17486481 is in full LD with the A allele of missense variant rs17408150 (669Q). Haplotype A-C encode multiple naturally occurring ERAP2 protein allotypes (see Table 1).

In humans, alternative splicing of the 19 exons of *ERAP2* is known to produce multiple transcripts that are under tight genetic control. Strong linkage disequilibrium between the SNVs in *ERAP2* results in the formation of two common haplotypes often referred to as haplotype “A” and “B” [6]. These haplotypes are best distinguished by alleles of the SNV (A-to-G) rs2248374 positioned inside the canonical 5’ splice site of exon 10 that by CRISPR-based mutation in genomic DNA was shown to control ERAP2 expression [20]. *ERAP2* mRNA and protein are produced by constitutive splicing of transcripts from the haplotype encoding the A allele of rs2248374 [6, 21]. In contrast, the G allele of rs2248374 breaks the motif in favour of a downstream motif which extends exon 10 so it includes multiple premature termination codons, leading to degradation of steady-state mRNA through nonsense-mediated mRNA decay (NMD) (Fig. 2). Individuals homozygous for the G allele of rs2248374 do not produce the 110 kDa ERAP2 protein, whereas individuals homozygous for the A allele express more ERAP2 protein than heterozygous individuals [6, 20–22].

Positive selection has targeted specific amino acid residues in *ERAP2* during mammalian evolution [23]. The ancestral *ERAP2* haplotype, found in contemporary chimpanzees, harbours the T allele of missense variant rs2549782 that encodes an asparagine at position 392 (392 N), which significantly broadens the substrate specificity of ERAP2 protein [24]. However, across modern human populations the ancestral T allele of rs2549782 is in full linkage (r^2^ = 1.0 in 1000 Genomes) with the G allele of rs2248374 (i.e., no enzyme expression) (Fig. 2). This indicates that there was selection against 392 N during human evolution, perhaps to narrow its specificity profile because of host-pathogen interactions. Similarly, missense variant rs75263594 at position 347 downstream of the GAMEN-motif would substitute a Threonine to Methionine and may affect peptide substrate recognition away from the N-terminus of ERAP2 (Efstratios Stratikos, Personal Communication), but the G allele of this variant is only found in haplotype B (D’ with rs2248374 = 1.0), which also precludes its translation into protein (Table 1). Perhaps similar selective mechanisms apply to another common variant rs17486481 located deep into intron 12–13 (allele frequency 5% in the European [EUR] superpopulation of the 1000 Genomes). The G allele of this variant introduces a donor splice site and produces an alternatively spliced transcript [25] (which we suggest calling “Haplotype C”) with an extended exon 12 that includes premature stop codons that also makes it prone to NMD. This is significant, because this splice variant is in full linkage with the A allele of missense variant rs17408150 (Leu669Gln) (Fig. 2), which is predicted to alter protein stability (global protein stability change prediction metric ΔΔGpred = 1.5, data from *ProtVar*). Consequently, mRNA transcribed from haplotypes expected to produce full-length ERAP2 (rs2248374-A), but which carry the A allele of rs17408150 (Gln669) will likely be destroyed by NMD. This is supported by the significant decrease in ERAP2 protein levels associated with the A-rs17408150 (Gln669) [26]. In summary, these two common splice region variants selectively remove transcripts that encode amino acids that significantly alter the function of the enzyme. While it remains uncertain if and what selective pressures operate against or in favour of specific ERAP2 protein isoforms, a deeper understanding of ERAP2 protein allotype diversity in the population may shed light on the evolutionary forces that shaped this gene.

## ERAP2 protein allotype diversity in the population

Allotype diversity between human populations may indicate potential adaptation to pathogenic species. In the 1000 Genomes Project dataset [27], the 10 most frequent missense variants in *ERAP2* in the global population form 10 haplotypes with a frequency of greater than 1% in one of the five major continental populations (Table 1). The ERAP2 allotype produced by the ancestral haplotype (which we will refer to as ERAP2*00) is very rare or absent in modern populations, while the most common haplotype (ERAP2*06 *A*) in the population is identical to the ancestral haplotype, but harbours the G allele of rs2248374 (i.e., precludes protein expression). There are five additional haplotypes predicted to produce enzymatically active ERAP2 protein allotypes which we will refer to as ERAP2*01-ERAP2*05, following previously suggested nomenclature [28], and 5 predicted to not produce full-length protein allotypes; ERAP2*06A-ERAP2*10 *A* (the suffix ‘A’ stands for ‘Aberrant’ expression where there is some doubt as to whether a protein is actually expressed [i.e., NMD under steady-state conditions]). The most common ERAP2 allotype (ERAP2*01) differs in amino acid sequence from the ancestral allotype at position 392. Additional amino acid positions determine other allotypes, such as ERAP2*03 (661 V) allotype that is common in Africa (2.3%) but absent in European and Asian populations. Also, ERAP2*02 (214 L) and ERAP2*05 (289 A) allotypes are common in Asia and rare (or absent) in other continental populations. To comprehensively map the landscape of allotype diversity in the general population, analysis of much larger sample sizes is needed (e.g., The UK Biobank includes data from 500,000 participants). Since ERAP2 is involved in a variety of human conditions [2], the polymorphic nature of the protein needs to be considered and allotype-specific study between pathogenic insult and immune response may help better understand the functional relation between ERAP2 allotypes and human disease.

**Table 1:**
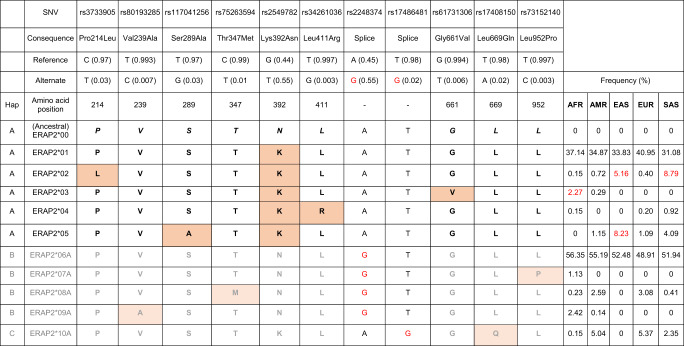
Haplotypes of common *ERAP2* missense variants and splice variants in the 1000 Genomes populations.

The *ERAP2* haplotypes were determined by using the phased genotype data from the 5 super populations of the 1000 Genomes. Haplotypes (Hap A) predicted to encode protein allotypes are indicated by bold black amino acid (single letter code). Haplotypes predicted to be subjected to nonsense mediated mRNA decay (Hap B and Hap C) controlled by splice variants (allele associated with alternative splicing are shown in red) are indicated in grey. The suffix ‘A’ (for ‘aberrant’, in accordance with HLA nomenclature) has been given to alternatively spliced transcripts due to the possibility of conditional expression or a lack of sufficient information to determine whether they are ‘null’ alleles. Non-ancestral AAs are shown as orange shaded boxes.

*AFR* African, *AMR* Ad Mixed American, *EAS* East Asian, *EUR* European, *SAS* South Asian.

## Keeping haplotypes in balance

The *ERAP2* sequence of chimpanzees and bonobos is >99% identical to that of humans, and the nucleotide sequence orthogonal to human exon 10 and the downstream splice region containing rs2248374 are identical (see Ensemble Genome Browser, accessed 31 July 2023) [29]. Intriguingly, these great apes are not polymorphic at the nucleotide position orthologous to rs2248374, and consequently lack the G allele that results in alternative splicing at exon 10 [6]. Therefore, the G allele may have been introduced after the split of our common ancestor during human evolution.

**Fig. 3:**
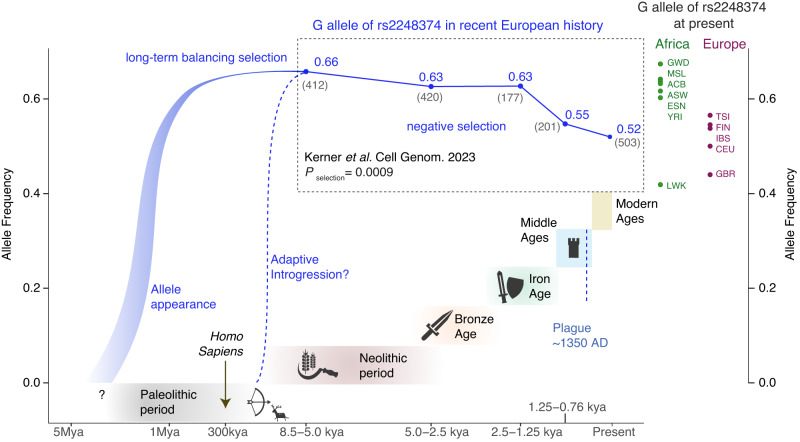
Graph showing the frequency of the G allele of rs2248374 throughout human history. The dark blue line indicates the trajectory of the frequency of the G allele of rs2248374 during human evolution until present. Historic allele frequency data and selection statistics (*P*_selection_) for rs2248374 were provided by *Kerner* et al. *Cell Genomics 2023* upon request (number of ancient DNA samples used to calculate the allele frequency in grey). The allele frequency for subpopulations of the African and European populations of the 1000 Genomes Project are also shown. Kya thousand years ago, Mya million years ago, CEU Utah residents with Northern and Western European ancestry, TSI = Toscani in Italia, GBR British in England and Scotland, FIN Finnish in Finland, IBS Iberian populations in Spain, YRI Yoruba in Ibadan, Nigeria, LWK Luhya in Webuye, Kenya, GWD Gambian in Western Division, The Gambia, MSL Mende in Sierra Leone, ESN Esan in Nigeria, ASW African Ancestry in Southwest US, ACB African Caribbean in Barbados.

Due to the scarce fossil records available during human evolution’s early stages, estimations of allelic introduction remain challenging. Also, complex human behaviours, such as migration and reticulation are difficult to address in evolutionary models [30]. Like much of present-day genetic variation in humans, the estimated time of introduction of the G allele of rs2248374 in homo sapiens traces back to a common ancestor of Neanderthals and humans (>700,000 years based on simulation-based prediction of modern human genome data) [31]. It may also be possible that *ERAP2* SNVs have been introduced through interbreeding (termed ‘introgression’) between archaic humans and ancestors of modern Eurasian populations (~50,000 years ago) [25] (Fig. 3). Adaptive introgression of gene alleles from Neanderthals to modern humans has been shown to be driven by host-RNA virus interactions [32], and the modelling of the *ERAP2* gene suggests Neanderthal-introgressed alleles, such as rs17409040 that is in full linkage with splice variant rs17486481 (i.e., Haplotype C could be a result of introgression) [25, 33, 34]. However, unlike variants that reside in haplotype C, the G allele of rs2248374 (i.e., haplotype B) is common in all global populations, including African populations considered devoid of archaic introgression, which indicates that rs2248374 probably arose in an early hominid ancestor and its allelic diversity in modern humans populations is perhaps result of continuous or recurrent contact between multiple very early African lineages [30].

Multiple independent studies have shown that the haplotypes of *ERAP2* have persisted over very long periods of time as the result of long-standing balancing selection (>Million years) [6, 35–37]. The process of balancing selection does not reduce genetic variation by selecting for alleles, but rather maintains multiple common alleles to conserve beneficial diversity within a population, in particular those involved in the co-evolutionary arms race between host and pathogens [37–39] In humans, *ERAP2* does not show signature of ancient balancing selection indicating that variants were likely introduced in the human lineage after the split from the chimpanzees [40]. However, the balancing selection signature of *ERAP2* is old and estimated at over more than a million years predating the split between humans and archaic hominins with the estimated time at which alleles have coalesced to a single common ancestor that lived up to ~1-4 Million years ago [6, 35, 36]. Evidence in support of long-term balancing selection are the very high polymorphic diversity of the *ERAP2* gene compared to the average protein-coding gene, and the maintenance of alleles of SNVs at intermediate frequency across the global human population [6, 35, 37, 38].

Alternatively spliced haplotypes of *ERAP2* are likely retained in populations either to maximise fitness or to confer an unknown adaptive benefit. Indeed, haplotype A has been associated with resistance against deadly infectious diseases [3, 4, 35]. Because haplotype A exhibits linkage to specific ERAP1 allotypes, we must consider the functional relationship between ERAP2 and its family member ERAP1 in evolutionary shaping of the *ERAP2* gene. Our laboratory reported in two collaborative studies that highly active ERAP1 allotypes harboured haplotype A of *ERAP2* less frequently than intermediate active ERAP1 allotypes, while intermediate active ERAP1 allotypes had the reverse pattern [41, 42]. ERAP1 and ERAP2 can form dimers and functionally complement each other in antigen processing [1, 2]. Mapping the functional ERAP2 allotypes of patients will therefore play an important role in better understanding disease mechanisms of conditions thought to be caused by ERAP1 rather than ERAP2 [5]. Another key question is why a haplotype B persists in humans despite the loss of functional ERAP2 protein (in steady state). Work by *Ye and co-workers* provided tantalising clues to answer this question by demonstrating that this presumed “null allele” encodes multiple truncated *ERAP2* isoforms that are selectively synthesised during influenza infection [43]. The G allele of rs2248374 has no effect on the expression of these isoforms since transcription starts at exon 9 (and lack GAMEN and HEXXHX motifs essential for enzymatic activity), and includes an in-frame start site at exon 11 [43]. There is also evidence that these haplotype B-derived *ERAP2* isoforms may be transcribed upon exposure to other microbial agents [44]. This has two significant implications; it proves that the widely accepted notion that a large group of individuals are “deficient” for ERAP2 may be prematurely concluded, and that alternatively spliced haplotypes may actually serve a non-redundant active immune-related purpose. Most functional research on ERAP2 is conducted in cell lines which after years of continuous cultivation are prone to profound chromosomal aberrations reported to also affect ERAP genes [45]. This emphasizes the importance of studying *ERAP2* under physiological inflammatory conditions (during infection and inflammation) and in primary tissues to answer outstanding questions regarding its biological functions. Note that a common SNV in the *ERAP1* gene also confers differential susceptibility to influenza infection, suggestive of similar pathogen-driven selection on the *ERAP1* gene [46]. These observations support that pathogenic pressure may have enforced selection on the *ERAP2* locus and that the maintenance of multiple functionally distinct haplotypes may indicate that ERAP2 function is non-redundant in humans.

## Recent genetic adaptation of *ERAP2*

While balanced alleles may be maintained for millions of years, they may change upon altering environments, such as historic migrations or changes in lifestyle [47]. In populations with profoundly different environments, such as between rainforest hunters-gatherers and farmer populations, we may expect evidence for pathogen-driven selection [48]. According to a study comparing a contemporary hunter-gatherer society with a neighbouring farmer population in Uganda that diverged more than 60,000 years ago, peripheral blood cells expressed significantly higher levels of *ERAP2* after stimulating viral sensory pathways in Hunter-gatherers as compared to agriculturalists, of which the former also had higher allele frequencies of SNVs that reside in Haplotype A [49]. Coincidentally, these hunter-gatherers exhibited higher titters of serum antibodies against DNA viruses. While this makes it tempting to attribute that selection of *ERAP2* variants may have been driven by differences in pathogenic exposure, the genetic selection statistics in this study did not support recent selection for *ERAP2* (in line with studies of other contemporary rainforest hunter-gatherer societies [48]), which indicates that this intriguing finding was likely due to the modest sample size inherent to studies of remote populations.

However, two recent studies in European populations demonstrate that the *ERAP2* alleles previously under long-term balancing selection were recently (<10,000 years ago) targeted by positive selection. A rapid positive selection for haplotype A was shown during the *bubonic plague* pandemic (*The Black Death*) caused by the bacterium *Yersinia pestis* that swept through Europe around 1350. Genotyping of remains of individuals before, during, and after the *Black Death* pandemic (caused by infection with *Yersinia Pestis*) revealed significant increase in frequency of the T allele of SNV rs2549794 (tags haplotype A) over the course and after the pandemic [3]. Although the reported increase in allele frequency in comparison to other studies remains topic of debate [50, 51], macrophages from present-day individuals that carried Haplotype A neutralised *Yersinia Pestis* more effectively in an in vitro analysis [3].

A recent large cohort of 2376 ancient and 503 modern European genomes sampled over the course of 10,000 years across the Neolithic period, the Bronze Age, the Iron Age, the Middle ages, and present-day Europeans tested for allelic frequency changes in SNVs across this historical period [52]. Data from this study revealed that the G allele of rs2248374 has evolved under negative selection over the last ~3000 years (estimated time of selection onset for the G allele of rs2248374 of 2866 [95% confidence interval = 317-8,747 years], based on data kindly provided by Kerner and co-workers [52]). This observation provides evidence for short-term natural selection in favour of haplotype A in very recent European history and is conceptually in line with the observations from the analysis of genomes during the *Black Death* pandemic. These two ‘longitudinal’ studies provide supporting evidence for very recent selection changes for long-standing balanced alleles of *ERAP2* as civilization spread across the European continent and encountered drastically altered environments. This local adaptation of *ERAP2* haplotypes is further supported by the observation that allele frequency of the G allele of rs2248374 during the Neolithic era in Europe is very close to the allele frequency in modern African populations (~0.65), as compared to modern European populations (~0.5)(with the exception of the African *Luyha* [LWK] population, which is genetically different from other African populations [53] (Fig. 3). While examples of genes under long-term balancing selection that show signatures of recent positive or negative selection have been described [47], more studies are needed to gain a deeper understanding of how *ERAP2* evolved over the course of human evolution. Haplotype-based analysis using missense variants that define naturally occurring ERAP2 allotypes (Table 1) may aid in understanding how and to what extent the biological functions of ERAP2 adapted to environmental pressure in recent history.

## ERAP2 haplotypes and autoimmunity

**Fig. 4:**
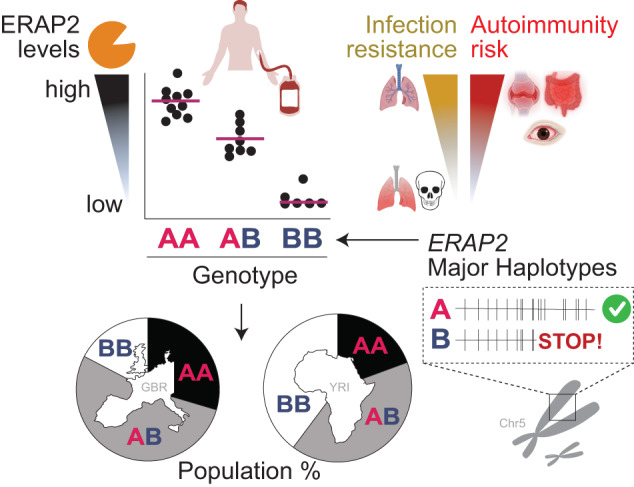
The impact of ERAP2 on human health & disease. A high level of (heritable) interindividual variation in ERAP2 is caused by the two major ERAP2 haplotypes A and B, which are controlled by alternative splicing mediated by the splice variant rs2248374 (Fig. 2). Furthermore, substantial differences in *ERAP2* genotype frequencies are observed between continental populations, such as between Africans and Europeans, and support genetic adaptation of *ERAP2* in human history. ERAP2 enhances resistance to respiratory infections but also increases the risk of autoimmune diseases. ERAP2’s role in health and disease may be better understood if we gain a deeper understanding of the function of ERAP2 protein allotype diversity across populations. The figure contains elements created with BioRender.com. GBR = British in England and Scotland, YRI = Yoruba in Ibadan, Nigeria; Genotype frequencies from GBR [= British in England and Scotland] and YRI [= Yoruba in Ibadan, Nigeria] populations of the 1000 Genomes Project.

Genetic variants that reside in haplotype A of the *ERAP2* gene predispose to a variety of severe inflammatory conditions, including among others, *Crohn’s* disease, ankylosing spondylitis, birdshot chorioretinopathy, and Juvenile idiopathic arthritis [5, 20]. The same alleles of autoimmune disease-risk variants have also been shown to provide increased protection against severe infections (a leading cause of death in the past) [3, 4, 52]. In a recent study of genetic data from large contemporary human biobanks, it was shown that SNV’s that tag haplotype A of *ERAP2* was associated with protection against respiratory infections, with increased ERAP2 expression in whole blood (i.e., haplotype A) being associated with protection against severe respiratory infection (such as Pneumonia and Covid-19), but with opposing effects on Crohn’s disease [4]. This *antagonistic pleiotropy* (i.e., alleles of SNVs that exhibit both beneficial and unfavourable effects) of genetic variants in *ERAP2* supports that the common haplotypes may have provided protection against infectious diseases in human history at the cost of a higher genetic risk for chronic inflammatory conditions in modern humans (Fig. 4). How haplotype A-encoded ERAP2 protein contributes to autoimmune conditions remains uncertain. Additional SNVs control *ERAP2* expression by mechanisms other than alternative splicing, and variants linked to autoimmune diseases also alter enhancer-promoter interactions of *ERAP2* leading to increased expression levels [20]. As a result, haplotype A may not be pathogenic in itself, but higher levels of ERAP2 associated with inflammatory processes may increase the risk of inflammatory diseases, although further studies are needed to determine whether these mechanisms are involved. The widespread adoption of single-cell transcriptomic profiling technologies will aid in capturing the biological regulation and dysfunction of ERAP2 in human pathologies. Due to the dramatic impact of common variants on ERAP2 expression levels, caution is warranted when interpreting data from studies with small sample sizes because genotype imbalances can strongly impact quantitative analyses of ERAP2 transcripts and proteins. Therefore, multi-*omic* (or “complementary”) single-cell technologies that also consider high-resolution genotyping in primary tissues should be used to investigate gene-by-environment interactions of ERAP2.

## In conclusion

According to current data, haplotypes of ERAP2 have opposing genetic association with chronic inflammation and infection. Susceptibility to autoimmune diseases in the human population may be related to *ERAP2* haplotypes that show evidence for natural selection in recent history because they are associated with resistance to infection as revealed by ancient genome studies. Further investigations of *ERAP2* allotype diversity in the modern human population combined with insights from evolutionary studies that model selective pressure on alleles will enhance our understanding of its function in immunity and potentially aid in the development of targeted therapies for autoimmune conditions and infectious diseases.

## Declaration of AI-assisted technologies in the writing process

During the preparation of this work the authors used Wordtune to improve readability. After using this tool, the authors reviewed and edited the content as needed and take full responsibility for the content of the publication.

## Data Availability

The datasets used in this review are available in the 1000 Genomes Project repository https://www.internationalgenome.org/ and allele frequencies for each population accessed via the Ensemble Genome Browser https://www.ensembl.org/.
